# IUC26686-93 Synergistic potential of cabozantinib and radiotherapy in advanced renal cell carcinoma by site of irradiation

**DOI:** 10.1093/oncolo/oyag286.025

**Published:** 2026-08-21

**Authors:** F Di Costanzo, A Bertocchi, M L Esposito, I S Parisi, A Federico, P A Zucali, L Formisano, C Oing, R Chandler, P Rescigno

**Affiliations:** Translational and Clinical Research Institute, Centre for Cancer, Newcastle University, Newcastle upon Tyne - United Kingdom; IRCCS Humanitas Research Hospital, Rozzano, Milan - Italy; Department of Clinical Medicine and Surgery, University of Naples Federico II, Naples - Italy; Interdisciplinary Department of Medicine, A. Moro University of Bari, Bari - Italy; IRCCS Humanitas Research Hospital, Rozzano, Milan - Italy; IRCCS Humanitas Research Hospital, Rozzano, Milan - Italy; Department of Clinical Medicine and Surgery, University of Naples Federico II, Naples - Italy; Translational and Clinical Research Institute, Centre for Cancer, Newcastle University, Newcastle upon Tyne - United Kingdom; Northern Centre for Cancer Care, Newcastle Hospitals NHS Foundation Trust, Newcastle upon Tyne - United Kingdom; Translational and Clinical Research Institute, Centre for Cancer, Newcastle University, Newcastle upon Tyne - United Kingdom

## Abstract

**Background:**

Cabozantinib is an established treatment for metastatic renal cell carcinoma (mRCC) and may be continued beyond limited radiological progression in selected patients. In oligoprogressive disease, site-directed radiotherapy (RT) may prolong systemic treatment benefit. We explored whether progression-free survival (PFS) differed according to the anatomical site of irradiated oligoprogression.

**Methods:**

This retrospective multicentre analysis included patients with mRCC receiving cabozantinib who underwent RT for oligoprogressive disease, defined as no more than three new or progressive lesions at restaging. To avoid double-counting patients treated at multiple anatomical sites, the irradiated site was analysed at the patient level using mutually exclusive categories: bone-only RT, brain-containing RT, visceral/nodal RT, and mixed RT, defined as bone plus visceral/nodal irradiation. Visceral/nodal sites included lung, thyroid, adrenal, lymph-node, and pancreatic metastases. PFS1 was defined from cabozantinib initiation to RT-treated oligoprogression. PFS2 was defined from RT to subsequent progression or death. PFS outcomes were summarized as median months with interquartile ranges and compared descriptively across groups.

**Results:**

Forty-one patients had available data on the irradiated metastatic site. Bone-only RT represented the most frequent category. Median PFS1 differed numerically across irradiated-site groups and was 13.2 months for bone-only RT, 7.4 months for brain-containing RT, 15.5 months for visceral/nodal RT, and 12.8 months for mixed bone plus visceral/nodal RT. Median PFS2 was 9.2 months, 6.6 months, 10.1 months, and 7.0 months, respectively (Table 1); notably, patients receiving RT to visceral/nodal oligoprogression showed the most favourable post-RT outcome.

**Conclusions:**

In this exploratory multicentre cohort, post-RT disease control during cabozantinib varied according to the anatomical site of irradiated oligoprogression. Visceral/nodal oligoprogression was enriched for prolonged post-RT disease control, suggesting that this subgroup may be particularly suitable for site-directed RT with continued cabozantinib. These hypothesis-generating findings require validation in larger prospective cohorts.

**IUC26686-93 Table 1:** PFS outcomes according to irradiated site category.

Irradiated site category (n)	Median PFS1, months (IQR)	Median PFS2, months (IQR)
**Bone-only RT (17)**	13.2 (7.6–18.1)	9.2 (5.8–10.2)
**Brain-containing RT (5)**	7.4 (4.0–17.3)	6.6 (4.2–9.1)
**Visceral/nodal RT (10)**	15.5 (11.3–21.3)	10.1 (7.3–12.5)
**Mixed RT (9)**	12.8 (10.2–14.6)	7.0 (4.0–8.3)

